# Necrotizing sialometaplasia—A self‐limiting condition which poses a diagnostic dilemma

**DOI:** 10.1002/ccr3.3931

**Published:** 2021-02-18

**Authors:** Abhishek Gupta, Anamika Priyadarshinee, Lavanya Nanjunda Rao, Anju Redhu, Kumari Sonam Jha

**Affiliations:** ^1^ Department of Oral Medicine and Radiology KIST Medical College and Teaching Hospital Kathmandu Nepal; ^2^ Department of Pathology KIST Medical College and Teaching Hospital Kathmandu Nepal; ^3^ Dental Unit Aster CMI Hospital Bengaluru India; ^4^ Department of Oral Medicine and Radiology PGIDS Rohtak UK; ^5^ Department of Oral Medicine and Radiology GDCRI Bangalore India

**Keywords:** carcinoma, necrotizing, oral ulcer, salivary gland diseases, sialometaplasia, squamous cell

## Abstract

Necrotizing sialometaplasia can make anyone very anxious about the lesion especially if they have habit of tobacco consumption. It requires a prompt diagnosis, counseling with assurance to patient and treatment.

## Graphical abstract

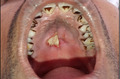

## 1 INTRODUCTION

Necrotizing sialometaplasia (NS) was first reported by Abrams et al^1^ in the year 1973, who had added this new clinical entity to the salivary gland pathology termed as “Necrotizing Sialometaplasia” which was the histological description of the lesion—as an inflammatory disease characterized by lobular necrosis by mucus escape reaction and by marked squamous metaplasia and pseudoepitheliomatous hyperplasia. This rare entity has been classified under inflammatory and reactive lesions of salivary gland diseases.^2^ This condition has been described as non‐neoplastic inflammatory salivary gland disease^2^ and benign, self‐limiting, reactive inflammatory disorder of salivary tissue. NS can resemble a malignancy and its misdiagnosis has resulted in unnecessary radical surgery.^3^ Mesa et al had reviewed approximately 10 000 oral biopsy specimens in the year 1984 and had revealed only three cases of necrotizing sialometaplasia, all of which had been misdiagnosed as other benign entities, representing only 0.03 percent of biopsied oral lesions.^4^ Shin et al had reviewed all biopsy materials taken from the oral cavity in a single institution in Korea from 2012 to 2018 and found 4 cases of NS out of 726.^5^ The largest series of cases of NS in the year 1991 reported average age at the diagnosis as 45.9 years, males outnumbered females by a ratio of 1.9:1, and whites outnumbered blacks by a ratio of 4.9:1.^6^ It may arise in any area containing salivary gland tissue. Classically, it involves the mucoserous glands of the hard palate. Other sites where it has been reported include nasal cavity, trachea, parotid gland, sublingual gland, submandibular gland, larynx, buccal mucosa, maxillary sinus, tongue, tonsil, and retro molar trigone.^7^ The etiology of this lesion is bizarre and controversial. The most commonly proposed and generally accepted etiology for NSM relates to ischemia.^6^ Other traumatic injuries, such as dental injection, blunt force trauma, denture wear, alcohol and tobacco use, and upper respiratory infections, have been implicated as potential predisposing factors.^7^ The exact pathophysiology of necrotizing sialometaplasia is unknown, but ischemia of the vasculature supplying the salivary gland lobules is the most widely accepted theory.^8^ Furthermore, two experimental studies were able to produce necrotizing sialometaplasia in submandibular and sublingual glands of rats by ligating the vasculature supplying these major salivary glands.^9^, ^10^ This lesion clinically presents as a deep ulcer. The size may range from 0.7 to 5.0 cm (average 1.8 cm) with sharply demarcated borders, often surrounded by an erythematous halo. The posterior hard palate is the most common site to be affected by NS and junction of the hard and soft palate being the second most common site. About two‐thirds of the palatal lesions are unilateral; however, midline, bilateral synchronous and metachronous lesions do occur.^11^, ^12^ The diagnosis of this lesion is based on the histological criteria proposed by Abrams et al,^1^ 1973—the presence of ischemic lobular necrosis of seromucous glands, squamous metaplasia of ducts and acini, preservation of intact lobular architecture despite necrosis and inflammation, and accumulation of necrotic debris in the adjacent lobules.^1^ The diagnosis is mainly based on the clinical features and histopathological analysis. Squamous cell carcinoma and mucoepidermoid carcinoma have been considered as the differential diagnosis in the previous literature. No specific treatment is required since lesion is self‐limiting and heals within the 6 to 8 weeks. This self‐limiting condition has not yet been reported in Nepal which led us to publish this rare entity.

## 2 CASE HISTORY/EXAMINATION

**FIGURE 1:**
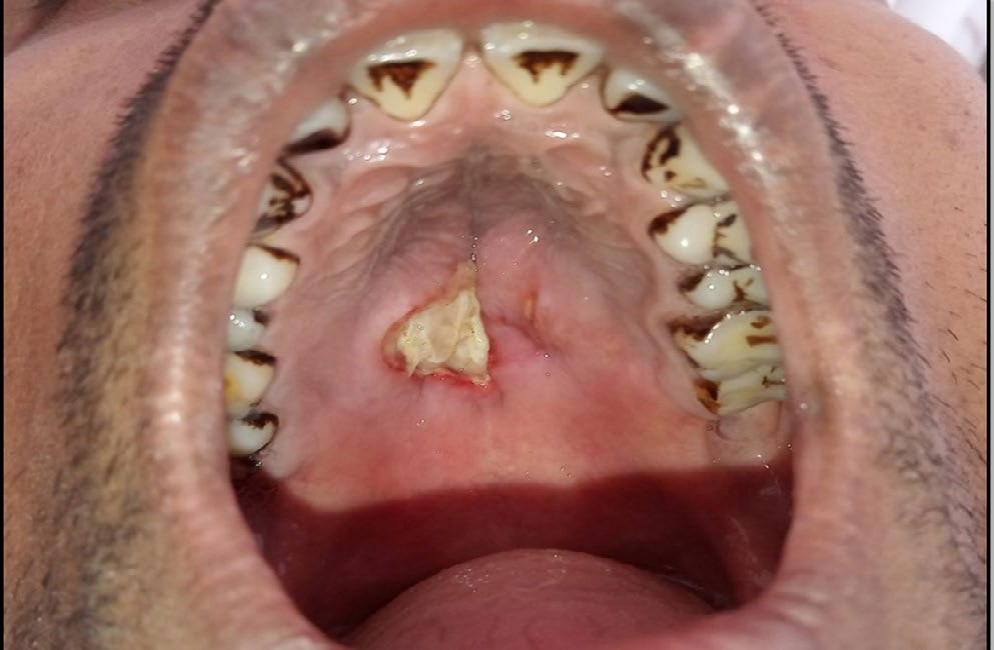
Intraoral view depicting the ulceration of the palatal region

A twenty‐six‐year‐old man reported to the department of oral medicine and radiology with the chief complaint of wound in the mid‐palatal region since 35 days (Figure 1). The patient noticed the ulcer which was initially a pea‐sized and gradually progressed to present size within the span of 15‐20 days and then remained of the approximately same size. Patient had the significant history of cigarette smoking for 10 pack‐years, which contributed to the anxiousness about the lesion due to cancerophobia. Patient had no significant medical history and past dental history, apart from prescription of different antibiotics and analgesics by previous dentists which could not improve his condition. There was no significant history of trauma. Extraoral examination was nonsignificant, and intraoral examination revealed generalized black stains on the coronal surface of the teeth and a solitary ulcer in the mid‐palatal region of size roughly 2 × 3 in its greatest dimension. The margin of an ulcer appeared rolled, erythematous and was covered with the yellowish slough. The lesion was nonindurated, slight tender on palpation. The regional lymph nodes were nonpalpable.

## 3 DIFFERENTIAL DIAGNOSIS, INVESTIGATIONS, AND TREATMENT

**FIGURE 2:**
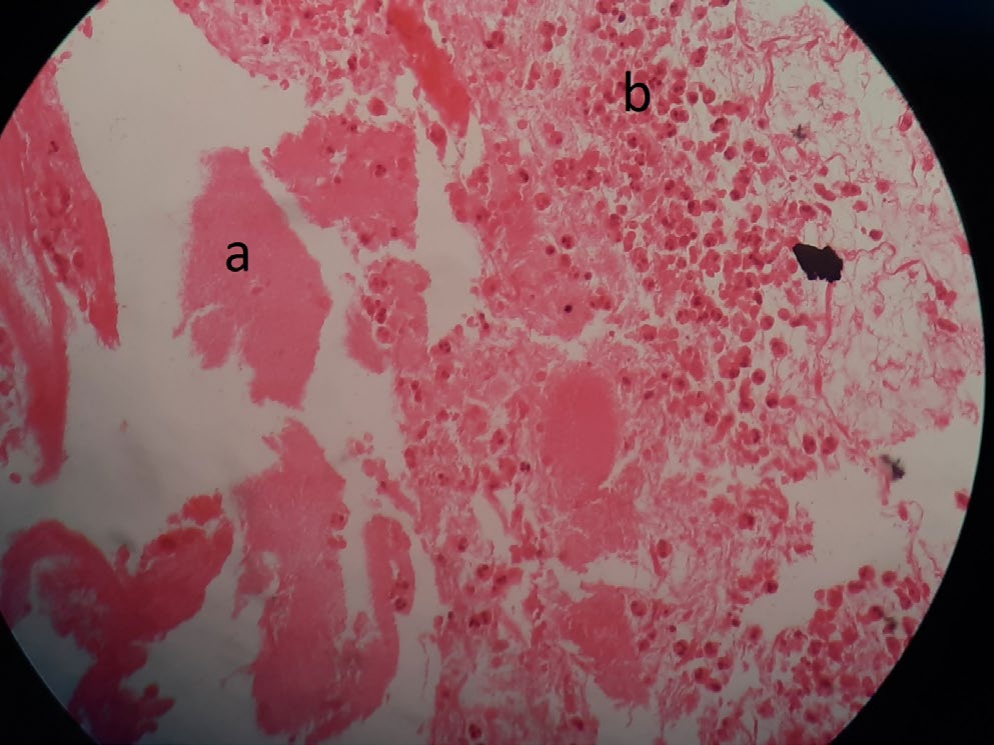
Fibrinoid necrosis (A) with sheet of neutrophils (B)

**FIGURE 3:**
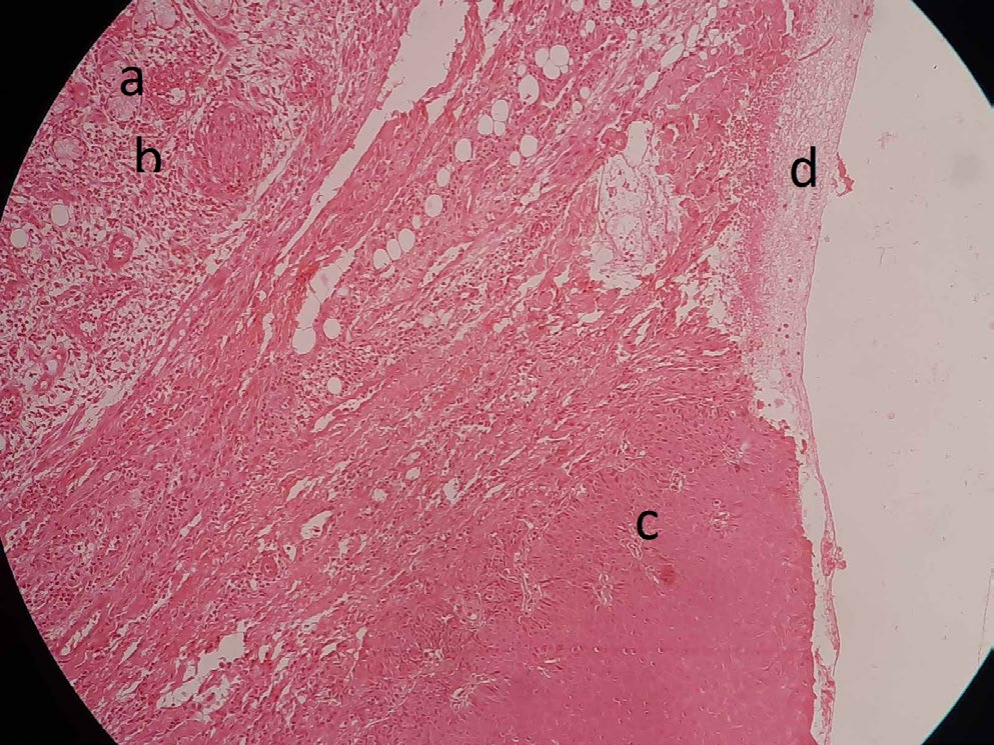
Few acini (A) surrounded by dense neutrophilic infiltration (B) are present in the left upper corner, hyperplastic stratified squamous epithelial lining (C) without atypia and dysplasia on the right lower corner. Ulcer with fibrinoid necrosis and inflammation (D)

A provisional diagnosis of malignant ulcer was given, and the differential diagnosis with necrotizing sialometaplasia was considered. The occlusal radiograph depicted normal bone architecture, and laboratory investigations were within normal limits. The patient was subjected to incisional biopsy using infiltration anesthesia. The histopathological examination revealed fibrinoid necrosis with sheet of neutrophils (Figure 2), and few acini surrounded by dense neutrophilic infiltration are present in the left upper corner, hyperplastic squamous epithelial lining present on the right lower corner (Figure 3). The final diagnosis of necrotizing metaplasia was arrived at based on clinical examination and histopathological analysis. The patient was subjected to symptomatic treatment, and debridement of lesion using normal saline was made. The patient was assured of the lesion being of benign nature and as tobacco being one of the causes, tobacco cessation counseling was also done.

## 4 OUTCOME AND FOLLOW‐UP

The lesion had completely healed after 15 days of the treatment. The patient is under regular follow‐up.

## 5 DISCUSSION

As the title says, this is the self‐limiting condition, and in our patient, the lesion healed within a span of 15 days. This posed us a diagnostic dilemma, since the patient was young, presented with the rapid onset of the ulceration with the positive history of exposure to carcinogenic products, was initially diagnosed as malignant ulcer. As a part of an investigation, an incisional biopsy was done which was surprisingly necrotizing sialometaplasia. This condition would have been considered as a differential diagnosis due to following factors such as typical location of its occurrence in the hard palate, gender of the patient and smoking being one of the predisposing factor. However, we missed out this lesion in the initial diagnosis probably because of its rarity, and not so prevalent in our country Nepal. This lesion was challenging for us during histopathological slide analysis since most of the features mimicked a malignancy. The careful examination of the histopathological features by an experienced pathologist led us to the correct diagnosis. Prior knowledge of the lesion and its features is very important to every clinician and every oral physician and oral pathologist so that misdiagnosis does not occur. After going through the literature based on this lesion, most of the case reports has reported this lesion in palatal region and very rarely in other location. The typical characteristics of this lesion are the presence of an ulceration with slough. Smoking can be considered as a probable etiological factor in this case since this is considered one among the etiology. However exact etiopathogenesis of how smoking causes necrotizing sialometaplasia has not yet been studied. Patient was subjected to routine laboratory investigation to rule out other conditions. Histopathological analysis using H&E staining is still considered as a gold standard, similarly done in our case. However, Rizkalla et al in his study used immunohistochemistry for identification of myoepithelial cells and CK7 expression which may help to distinguish NSM from its mimics,^13^ and this lesion usually does not require any specific treatment; however, symptomatic and conservative management is required.

## CONFLICT OF INTEREST

The authors declare that there is no conflict of interests regarding the publication of this paper.

## AUTHOR CONTRIBUTIONS

AG: performed clinical diagnosis and selection of case, done literature search, and edited and reviewed the manuscript. AP: provided with the histopathological interpretations and photomicrograph, and edited and reviewed the manuscript. LNR: written the manuscript at initial phase, and edited and reviewed the manuscript. AR and KSJ: edited and reviewed the manuscript for final manuscript preparation.

## ETHICAL APPROVAL

Written consent was taken from the patient.

## Data Availability

The data are available with the correspondence author and can be availed on request.
